# Persistent SARS-CoV-2 antigen presence in multiple organs of a naturally infected cat from Brazil

**DOI:** 10.1590/1678-9199-JVATITD-2021-0074

**Published:** 2022-03-07

**Authors:** Samar Afif Jarrah, Louise Bach Kmetiuk, Otávio Valério de Carvalho, Alessandra Tammy Hayakawa Ito de Sousa, Valeria Regia Franco Souza, Luciano Nakazato, Edson Moleta Colodel, Andrea Pires dos Santos, Christina Pettan-Brewer, Rosane Christine Hahn, Renata Dezengrini Slhessarenko, Daniel Guimarães Ubiali, Asheley Henrique Barbosa Pereira, Helio Autran de Morais, Alexander Welker Biondo, Valéria Dutra

**Affiliations:** 1 Department of Veterinary Medicine, College of Veterinary Medicine, Federal University of Mato Grosso, Cuiabá, MG, Brazil.; 2 Graduate College of Molecular Biology, Federal University of Paraná, Curitiba, PR, Brazil.; 3 Department of Comparative Pathobiology, College of Veterinary Medicine, Purdue University, West Lafayette, IN, USA.; 4 Department of Comparative Medicine, School of Medicine, University of Washington, Seattle, Washington, USA.; 5 Medical Science Faculty, Federal University of Mato Grosso, Cuiabá, MT, Brazil.; 6 Júlio Muller University Hospital, Federal University of Mato Grosso, Cuiabá, MT, Brazil.; 7 Department of Epidemiology and Public Health, Veterinary Institute, Federal Rural University of Rio de Janeiro, Seropedica, RJ, Brazil.; 8 Department of Clinical Sciences, Oregon State University, Corvallis, OR, United States.; 9 Department of Veterinary Medicine, Federal University of Paraná, Curitiba, PR, Brazil.

**Keywords:** Coronavirus, SARS-CoV-2, Pets, Cats, Disease transmission

## Abstract

**Background:**

Severe acute respiratory syndrome coronavirus 2 (SARS-CoV-2) is the etiological agent of the disease coronavirus 2019 (COVID-19) in humans. SARS-CoV-2 has been identified in cats with or without clinical signs.

**Case presentation:**

We describe the pathological and molecular findings in a six-month-old asymptomatic cat with SARS-CoV-2 infection from Brazil, belonging to a human family with COVID-19 cases. The pool of nasopharynx and oropharynx swabs at day zero tested positive by RT-qPCR for SARS-CoV-2. No amplification resulted from molecular testing performed on days 7 and 14. The cat was hit by a car and died 43 days after the molecular diagnosis. Immunohistochemistry at *post-mortem* examination demonstrated nucleocapsid protein in samples from the lungs, kidneys, nasal conchae, trachea, intestine, brain and spleen.

**Conclusion:**

The present study has highlighted the possibility that viral antigens can be detected by immunohistochemistry in multiple organs six weeks after infection, although the same tissues tested negative by RT-PCR.

## Background

The novel coronavirus known as severe acute respiratory syndrome coronavirus or SARS-CoV-2 is a zoonotic pathogen first detected in humans in December 2019 in China [1]. SARS-CoV-2 has been identified in cats with or without clinical signs. The majority of cases were from cats living in close contact with infected humans [2]. In March 2020, the first cat with SARS-CoV-2 infection was identified in Hong Kong from nasopharyngeal and oropharyngeal swabs, and fecal samples. That cat was clinically healthy and screened because its owner was hospitalized [3]. 

In cats, SARS-CoV-2 penetrates the cell by binding to the angiotensin-converting enzyme 2 receptor (ACE2). The viral spike protein interacts with the ACE2 receptor, whose feline and human versions are highly homologous [4]. Under experimental conditions, domestic cats intranasally inoculated with SARS-CoV-2 have shown no clinical signs but shed viral RNA in feces and nasopharyngeal secretions [5]. Cats developed neutralizing IgG antibodies 24 days after intranasal inoculation [5]. Inoculated cats successfully transmitted the virus to cohoused cats [5,6]. Young cats showed more severe lesions in the trachea and lungs and may be more susceptible to the virus [5]. 

Transmission from cats to humans has not been previously identified. The ability of cats to transmit the virus to other cats experimentally highlights the potential for transmission to humans and other cats in the household. Cats have been considered potential sources of transmission of SARS-CoV-2 among mink farms in The Netherlands [6]. As part of an ongoing project to evaluate the impact of SARS-CoV-2 in pets exposed to infected humans in Brazil, this study aims to describe SARS-CoV-2 infection in a six-month-old, apparently healthy female indoor cat (*Felis catus*) living with a human family that had several members infected with COVID-19.

## Case presentation

This study was approved by the Ethics Committee for Animal Use at the Federal University of Mato Grosso (protocol 23108.043344/2020-62). 

In the city of Cuiabá (15°35′45″S, 56°05′49″W), the capital of Mato Grosso state in central-western Brazil, after a family gathering on September 19^th^, 2020, nine relatives were diagnosed with SARS-CoV-2 by RT-PCR on September 28th, with two people hospitalized and Cycle Thresholds (CT) of RT-PCR varying from 18 to 21. On October 5^th^, 2020 (day zero) nasopharyngeal and oropharyngeal swabs were taken and tested for SARS-CoV-2 by RT-PCR from a six-month-old female indoor cat and an adult dog, both asymptomatic, living in a household with three human cohabitants, all infected, and with nine infected humans in total after the gathering.

The cat underwent clinical evaluation in the Veterinary Teaching Hospital at the Federal University of Mato Grosso, and was asymptomatic at days zero, 7, and 14. In addition, the cat was given not only prophylaxis against ectoparasites, but also deworming and vaccination. The pool of nasopharynx and oropharynx swabs at day zero tested positive by RT-qPCR for SARS-CoV-2 by two different protocols (ORF-1a CT = 29.3, N gene CT = 31.5 and N1 CT = 35.3 and N2 CT = 35.1). No amplification resulted from molecular testing performed on days 7 and 14. Housekeeping genes successfully amplified in all samples.

The first protocol involved viral RNA extraction using a commercially available kit (MagMAX™ Viral/Pathogen Nucleic Acid Isolation Kit, Applied Biosystems, Foster City, CA, USA), and detected by RT-PCR using a commercial kit (TaqPath COVID-19 CE-IVD RT-PCR Kit, Applied Biosystems, Foster City, CA, USA), that targets the ORF-1a and nucleocapsid genes in different conserved genomic regions of SARS-CoV-2. The second protocol included total RNA extraction carried out with a commercial kit (Maxwell® RSC simplyRNA Tissue Kit, Promega Co, Madison, WI, USA), and SARS-CoV-2 RT-qPCR via the commercial kit (GoTaq® 1-Step RT-qPCR System, Promega Co, Madison, WI, USA) and the commercial kit (2019-nCoV CDC EUA RUO Kit, Integrated DNA Technologies, Newark, NJ, USA), targeting two regions (N1 and N2) of the nucleocapsid (N) gene. The feline β-actin gene was used as the internal control gene.

In addition, oligonucleotide targets of other respiratory and non-respiratory pathogens have been tested to achieve differential diagnosis, as previously described, including Feline Immunodeficiency Virus, Feline Leukemia Virus, Feline Coronavirus, Feline Herpesvirus, Feline calicivirus and the bacterium *Chlamydophila felis*.

On November 18th, the cat escaped outdoors and died after being hit by a car. Samples of brain, lung lobes, nasal conchae, trachea, lymph nodes, spleen, skin, kidneys, liver, stomach, small and large intestines, pancreas and heart were collected and fixed by immersion in 10% buffered formalin. The owners gave their consent for the necroscopy of their cat and for all the analyses performed. Blocks were cut into 3 μm slices and mounted onto silane-coated microscope slides, stained with hematoxylin and eosin (HE) and Masson’s Trichrome (MT) and evaluated by light microscopy. Formalin-fixed paraffin-embedded tissues were tested by immunohistochemistry [7].

Antigen retrieval was performed with citrate buffer (10 mM, pH 6.0) at 96º C for 20 min. The sections were then incubated in methanol:H_2_O_2_ solution (97%:3%) for 30 min to block nonspecific binding. The sections were set at 37°C for 3 hours with primary anti-SARS-CoV-2 nucleocapsid protein (NP) antibody (Catalog no. NB100-56576, Novus Biologicals, Centennial, CO, USA) by 1:500 dilutions and phosphate buffer saline (PBS) as the negative control. Lung and kidney sections of a SARS-CoV-2 non-infected cat were employed as an additional negative control. Lung tissue with compatible lesions and SARS-CoV-2 RT-PCR positive test from a human was used as the positive control. A streptavidin-biotin-peroxidase commercial kit (LSAB + System HRP, Agilent Technologies, Santa Clara, CA, USA) was employed as the detection system. Peroxidase activity was visualized with 3,3-diaminobenzidine chromogen (DAB + Substrate Chromogen System, DakoCytomation, Glostrup, Denmark). The sections were counterstained with hematoxylin, cover-slipped, and then viewed with a light microscope.

**Figure 1.**
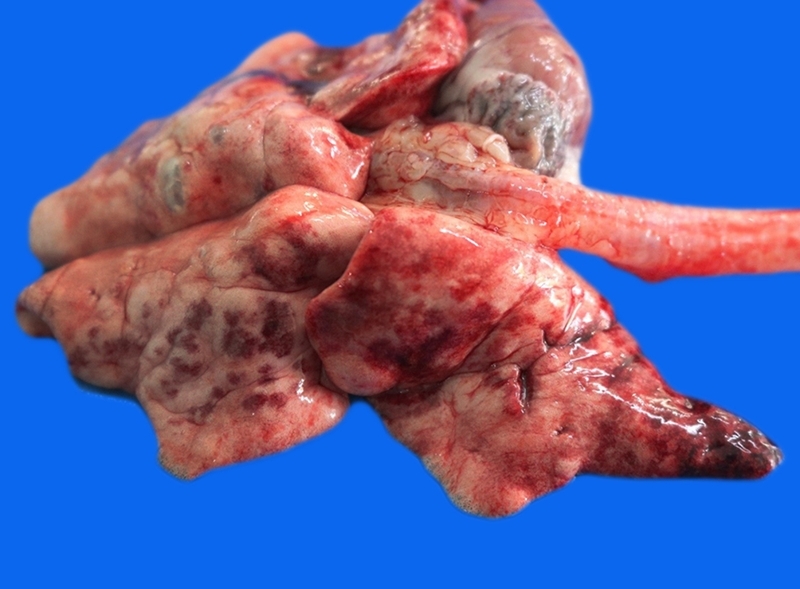
Pathological findings in a cat (*Felis catus*) with SARS-CoV-2: at necropsy, lungs present multifocal hemorrhages.

At necropsy, findings included traumatic brain injury with bone fracture of the skull, brain hemorrhage, and diffuse blood aspiration in the lung (Figure 1), likely due to the car accident. At histology, extensive multifocal hemorrhage in the brain and lungs were associated with mechanical trauma.

**Figure 2.**
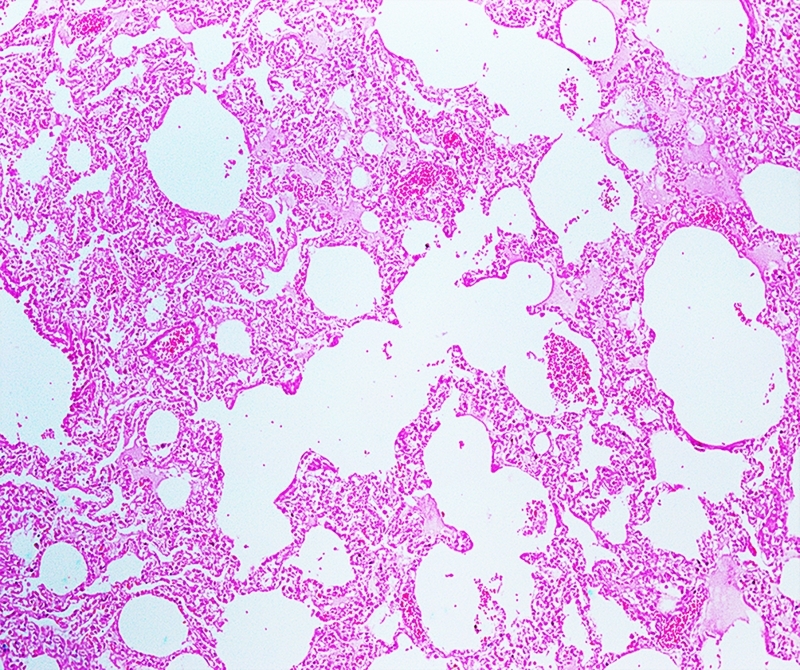
Pathological findings in a cat (*Felis catus*) with SARS-CoV-2: lung section with multifocal thickened alveolar septa with a moderate number of macrophages and edema. HE Obj. 20x.

**Figure 3.**
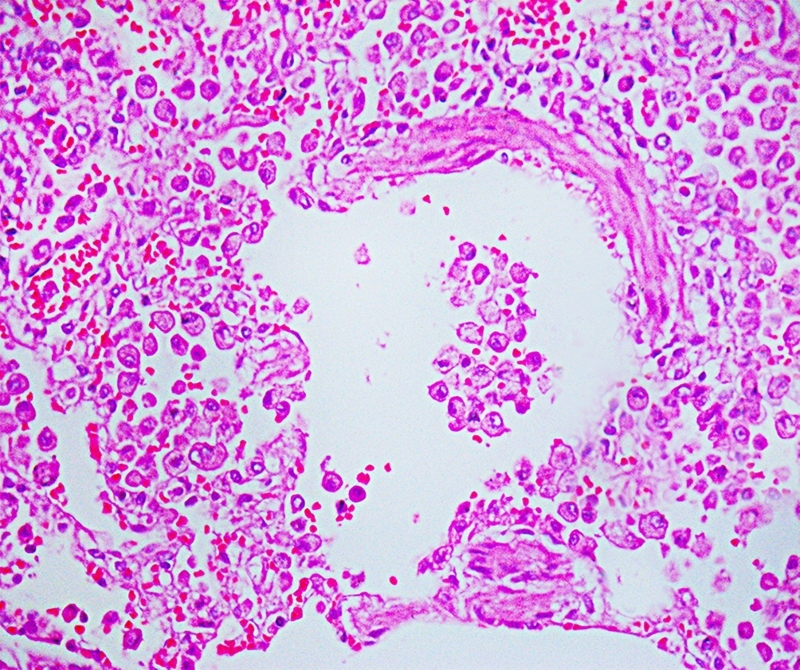
Pathological findings in a cat (*Felis catus*) with SARS-CoV-2: the walls of alveolar septa are thickened by fibroblastic proliferation and marked infiltration of alveolar macrophages. HE. Obj. 40x.

**Figure 4.**
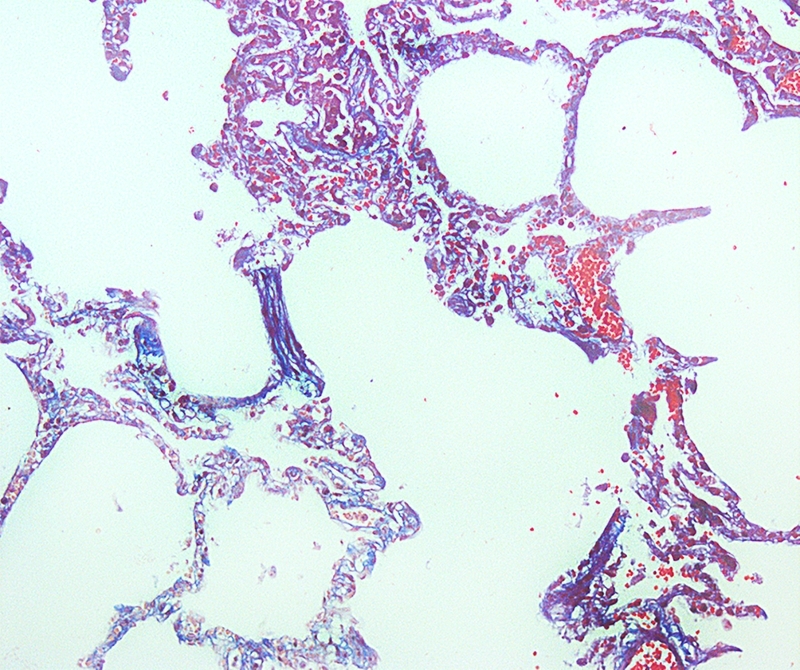
Pathological findings in a cat (*Felis catus*) with SARS-CoV-2: multifocal proliferation of fibroblastic tissue and collagen in the alveolar septa. Masson's Trichrome. Obj. 20x.

Lymphohistiocytic interstitial pneumonia represented about 10% of lung parenchyma (Figure 2). Occasional reactive macrophages in the alveolar or bronchiolar lumen (Figure 3), multifocal loss of type 1 and type 2 pneumocytes, and cellular debris within the alveoli were also observed. MT-stained pulmonary parenchyma exhibited multifocal fibroplasia (Figure 4), mainly at the distal end of alveolar ducts. Other consistent findings included hyperemia of alveolar septa and alveolar and interlobular edema. Lung lesions were likely related to SARS-CoV-2 infection.

**Figure 5.**
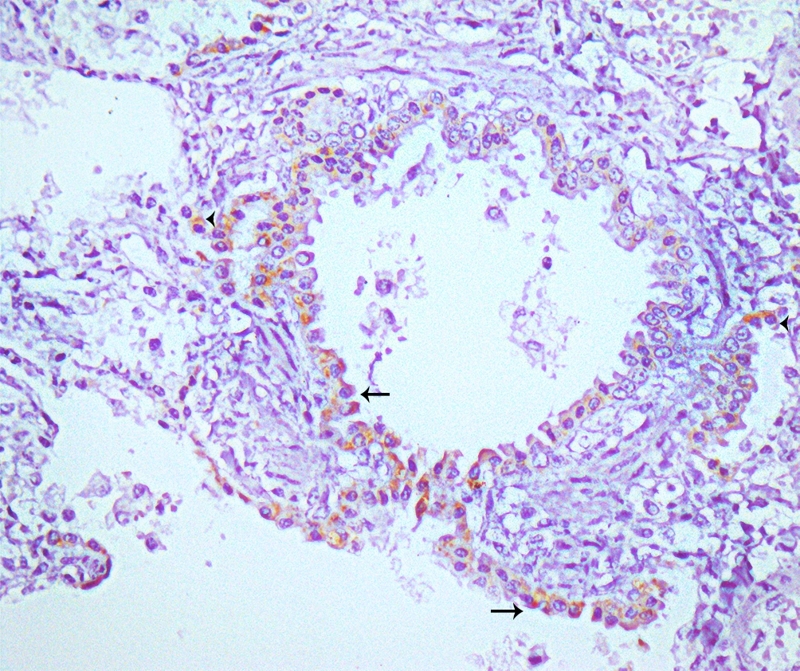
Immunohistochemical findings in a cat (*Felis catus*) infected with SARS-CoV-2: cytoplasmic immunolabeling in bronchiole respiratory epithelium (arrow) and interstitial macrophages (arrowhead) of anti-SARS-CoV nucleocapsid protein. Obj. 40X.

**Figure 6.**
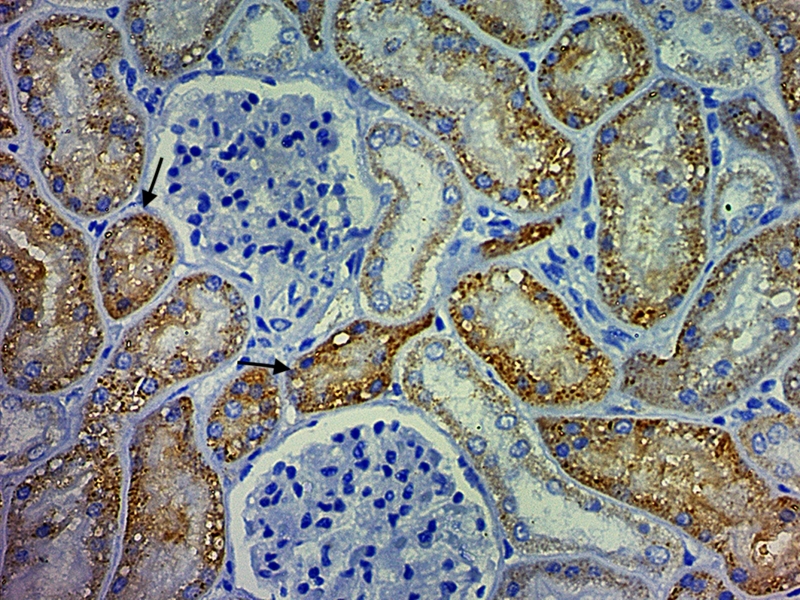
Immunohistochemical findings in a cat (*Felis catus*) infected with SARS-CoV-2: strong cytoplasmic immunolabeling of anti-SARS-CoV nucleocapsid protein in convoluted tubules of the kidney. IHC. Obj. 40x.

Strong immunelabeling was observed in the respiratory epithelium of the nasal conchae, trachea, bronchi, bronchiole (Figure 5), and the serous cells of tracheobronchial submucosal glands. The antigen expression observed in the lung also showed positivity in type 1 and 2 pneumocytes, and alveolar macrophages. Strong immunolabeling was also observed in epithelial cells of the kidney marking convoluted tubules (Figure 6), collector ductus, and intestinal gland epithelium. Strong positive immunolabeling of endothelial cells was found in the lungs and brain. Mild immunolabeling was seen in the splenic macrophages and arteriolar endothelium. Heart, liver, lymph node, stomach, and pancreas were all negative. Respiratory and non-respiratory etiological agents were tested to rule out coinfection and confounding pathogens. There was no molecular amplification for the presence of respiratory and non-respiratory etiological agents. Furthermore, no molecular amplification was observed in lung samples for SARS-CoV-2 (N1 and N2 targets), Feline Herpesvirus 1 (FHV-1), Feline Calicivirus (FCV), *Bordetella bronchiseptica*, *Chlamydophila felis* or *Mycoplasma felis* in standard PCR testing. Also, no molecular amplification was observed in spleen and kidney pool samples for *Mycoplasma felis*, Feline coronavirus (FCoV), *Cytauxzoon* spp., *Bartonella* spp., *Ehrlichia* spp., *Anaplasma* spp., *Candidatus Mycoplasma haemominutum* or *Candidatus Mycoplasma turicensis* in standard PCR testing. Additionally, the cat was seronegative for Feline immunodeficiency virus (FIV) and Feline leukemia virus (FeLV) by rapid FIV/FeLV test. 

## Discussion

SARS-CoV-2 has been reported as a causative agent of chronic lethal respiratory disease in cats [8]. The cat described herein most likely became infected by SARS-CoV-2 from the infected owners in the household [2]. Cats have been reportedly susceptible to SARS-CoV-2 infection, and viral RNA has been detected in nasal turbinates, soft palates, tracheas, lungs, and small intestines in young cats 3 to 6 days post-viral challenge [5].

Surprisingly, the asymptomatic cat reported in the current study presented chronic pulmonary lesions of SARS-CoV-2, with persistent antigens in the lungs six weeks after the first molecular detection. A previous study has shown chronic lung sequelae in cats inoculated with SARS-CoV-2 one month after viral infection recovery [8]. The anti-SARS-CoV nucleocapsid protein used as a primary antibody has been applied for SARS-CoV-2 investigations in the caecum and mediastinal lymph nodes [7] and central nervous system [9] of nonhuman primates and in the human placenta [10]. In the present study, no antigen was detected in the pancreas, heart, liver or lymph nodes. SARS-CoV-2 virus antigen was found in nasal turbinates, nasal swab, and mesenteric lymph node of a symptomatic, naturally infected cat, after humane euthanasia due to unrelated comorbidities [11]. 

In human COVID-19 cases, diffuse alveolar damage may be divided into 2 phases: the acute/exudative phase, during the first week after pulmonary injury, and the second, the organizing/proliferative phase [12]. The present results suggest that cats develop fibroplasia after alveolar damage due to previous SARS-CoV-2 infection [8,13], which may be classified as the late fibroplasia phase, regardless of molecular SARS-CoV-2 detection. This outcome has differed from idiopathic pulmonary fibrosis in adult cats (10 to 14-year), which were negative for the presence of multiple viral etiological agents [14]. Comparatively, human cases of COVID-19 have shown alveolar regeneration initiated by the 38^th^ day after clinical onset [15].

Negative RT-PCR results on subsequent oropharynx swabs did not detect viral shedding during this period (days 7 and 14). Despite the strong antigenic immunolabeling of anti-SARS nucleocapsid protein in multiple organs, non-detection of SARS-CoV-2 amplification by RT-PCR in tissues may indicate no viral replication or virus degradation at the time (day 43). A detection discrepancy between RT-qPCR and IHQ in renal tissue has been previously described [16] and may be due to a difference in abundance of target genes based on subgenomic (E and ORF7) transcripts in the late clinical phase of infection [17]. The presence of antigens in the absence of replicative virus has been previously found in lungs of macaques, baboons and marmosets experimentally inoculated with the SARS-CoV-2 virus [18]. Further studies should be performed to pinpoint the endpoint of viral replication in cats.

The cat in the current report presented a subacute systemic infection affecting mostly respiratory and renal epithelial cells, and endothelial cells of the brain and spleen. Although SARS-CoV-2 antigens were surprisingly found in the cat’s kidney, a retrospective study of 85 human cases of COVID-19 has shown NP antigen accumulated in kidney tubules by immunohistochemistry, associated with acute kidney failure in 23/85 (27.06%) individuals [19]. 

*In situ* assays, such as immunohistochemistry, have been essential diagnostic tools in SARS-Cov-2 cases [20]. The cat herein showed an intense antigen expression in respiratory epithelium, similar to SARS-CoV-2 infections in other species such as humans [12]. The expression of the anti-nucleocapsid protein in the large intestine, like other organs, is presumable due to high expression of angiotensin-converting enzyme 2 (AEC2) receptors in epithelial crypt cells of cats [21]. Immunolabeling of serous cells of tracheobronchial glands has been reported in cats experimentally infected with SARS-CoV [22] and SARS-CoV-2 [23], suggesting a similar pathogenesis mechanism. SARS-CoV-2 viral protein was also previously found mostly in lungs of humans, associated with histopathological manifestation including characteristic inflammatory response of acute respiratory distress syndrome (ARDS) [24]. This previous study was a systematic review of histopathological and clinicopathologic findings of SARS-CoV-2 infection in 58 studies reporting on 662 human patients. Future studies of SARS-CoV-2 in cats should be conducted to fully establish the origin of extensive lung lesions, and potential association with characteristic inflammatory response.

## Conclusion

In conclusion, the present study has highlighted the possibility that SARS-CoV-2 antigens may be detected in multiple organs, despite testing negative by real-time RT-PCR. To the best of the authors’ knowledge, this is the first naturally infected cat assessed by immunohistochemistry in multiple organs after viral recovery. Further studies should be conducted to fully establish the origin of extensive lung lesions, how long SARS-CoV-2 antigens remain detectable in cat tissues, and whether such persistence may cause impairment or injury of multiple organs over time.
